# Association between geriatric nutritional risk index and cognitive function in older adults with/without chronic kidney disease

**DOI:** 10.1002/brb3.70015

**Published:** 2024-09-11

**Authors:** Shan Wang, Jiajia Zhang, Jiaru Zhuang, Yuan Wang, Dewu Xu, Yibo Wu

**Affiliations:** ^1^ Obstetrics, Gynecology and Reproduction Research Affiliated Hospital of Jiangnan University Wuxi P. R. China; ^2^ Department of Medical Education Affiliated Hospital of Jiangnan University Wuxi P. R. China

**Keywords:** cognitive function, elderly, geriatric nutritional risk index, NHANES

## Abstract

**Background:**

Cognitive impairment is highly prevalent among patients with chronic kidney disease, who face an increased risk of cognitive decline. The aim of this study was to investigate the relationship between the Geriatric Nutritional Risk Index (GNRI) and cognitive function in older individuals, both with and without chronic kidney disease (CKD).

**Methods:**

In this study, we analyzed data from 2728 participants in the 2011–2014 National Health and Nutrition Examination Survey (NHANES). Cognitive function was measured using the Consortium to Establish a Registry for the Alzheimer's Disease Word Learning subtest (CERAD W‐L), the animal fluency test (AFT), the digit symbol substitution test (DSST), and the global cognitive *z*‐score. The GNRI, representing whole‐body nutritional status, was calculated based on serum albumin, body weight, and ideal body weight. We employed weighted multiple linear regression analyses and subgroup analyses to assess the independent association of GNRI with cognitive function in CKD and non‐CKD populations. Smoothing techniques were used to fit curves, and interaction tests were used to assess the robustness and specificity of the findings.

**Results:**

Our analyses revealed a significant positive association between higher GNRI levels and cognitive function in the older US population (for global *z*‐score: *β* = 0.01; 95% confidence interval [CI] = 0.00, 0.01). This association remained consistent across various subgroup analyses, including those for different gender groups, age groups, smoking statuses, diabetes statuses, hypertension statuses, individuals with a BMI below 25, individuals who consumed alcohol, and non‐Hispanic white individuals. Smoothed curve‐fitting analyses indicated that the GNRI was linearly related to cognitive function. No statistically significant interactions were detected among these variables.

**Conclusion:**

Our findings emphasize the positive association between GNRI and cognitive health in individuals with or without CKD, especially when combined with other risk factors. Consequently, enhancing the nutritional status of the elderly may serve as a viable strategy to thwart the onset of cognitive decline.

## Graphical abstract

Lower levels of the Geriatric Nutritional Risk Index (GNRI) are significantly associated with cognitive decline in the elderly population in the United States. GNRI may represent a new indicator for assessing cognitive health risks in the elderly. As a tool for evaluating the nutritional status of older adults, GNRI is cost‐effective and easily obtainable in clinical practice.

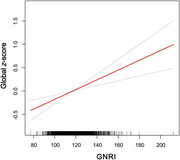

## 1 INTRODUCTION

Chronic kidney disease (CKD) has become one of the major diseases threatening human health. According to statistics, more than 850 million people globally suffer from CKD, which resulted in over 3.1 million deaths in 2019 (Jager et al., 2019; Kovesdy, 2022).

As global populations age, cognitive health emerges as a crucial public health challenge (Fishman, 2017). Cognitive abilities, such as memory, attention, reasoning, and language, are vital for daily functioning. However, cognitive impairments, spanning from mild cognitive impairment (MCI) to profound dementia varieties like Alzheimer's disease, significantly affect memory, judgment, decision‐making abilities, and daily life activities (Albert et al., 2011). These cognitive deficits not only impose significant economic and social burdens on families and communities but are also projected to account for 11% of global healthcare spending by 2025 (Hendriks et al., 2021; Prince et al., 2013). In the absence of a definitive cure for dementia, it is crucial to identify and implement effective preventative measures against cognitive deterioration.

Cognitive decline represents a frequently neglected complication associated with chronic kidney disease. In recent years, the relationship between nutrition and cognitive function in older adults has attracted considerable interest from researchers, particularly the potential impact of overall dietary patterns rather than single nutrients on cognitive function (Valls‐Pedret et al., 2015). This trend reflects a growing interest in how to maintain cognitive health through improved dietary habits (Barnes et al., 2023). Although such studies are increasing, research on whether specific nutrients or nutritional interventions can reverse the deleterious effects on cognition and help improve outcomes for older adults and people with Alzheimer's disease remains limited (Fadó et al., 2022).

Recent developments in nutritional assessment tools, such as the Geriatric Nutritional Risk Index (GNRI), provide a means of assessing nutritional status and predicting health outcomes in older adults (Bouillanne et al., 2005). The GNRI, which evaluates nutritional risk using simple measurements like weight, ideal body weight, and serum albumin levels, has been strongly associated with the prognosis of physical health conditions including diabetes (Shen et al., 2023), sarcopenia (Shiroma et al., 2023), osteoporosis (W. Huang et al., 2022), and depression (Li et al., 2024). Due to its ease of use and ability to make objective calculations based on available data, the GNRI is increasingly being utilized in clinical practice. By using tools like the GNRI, physicians and dietitians can better identify older adults whose malnutrition may affect their disease prognosis and develop interventions accordingly. Thus, this study sought to examine the correlation between the GNRI and cognitive abilities in older individuals with or without CKD, utilizing data from the 2011–2014 NHANES. The goal is to provide insights that could lead to more impactful methods to enhance the well‐being of the elderly through nutritional assistance.

## 2 METHOD

### 2.1 Study population and design

**FIGURE 1:**
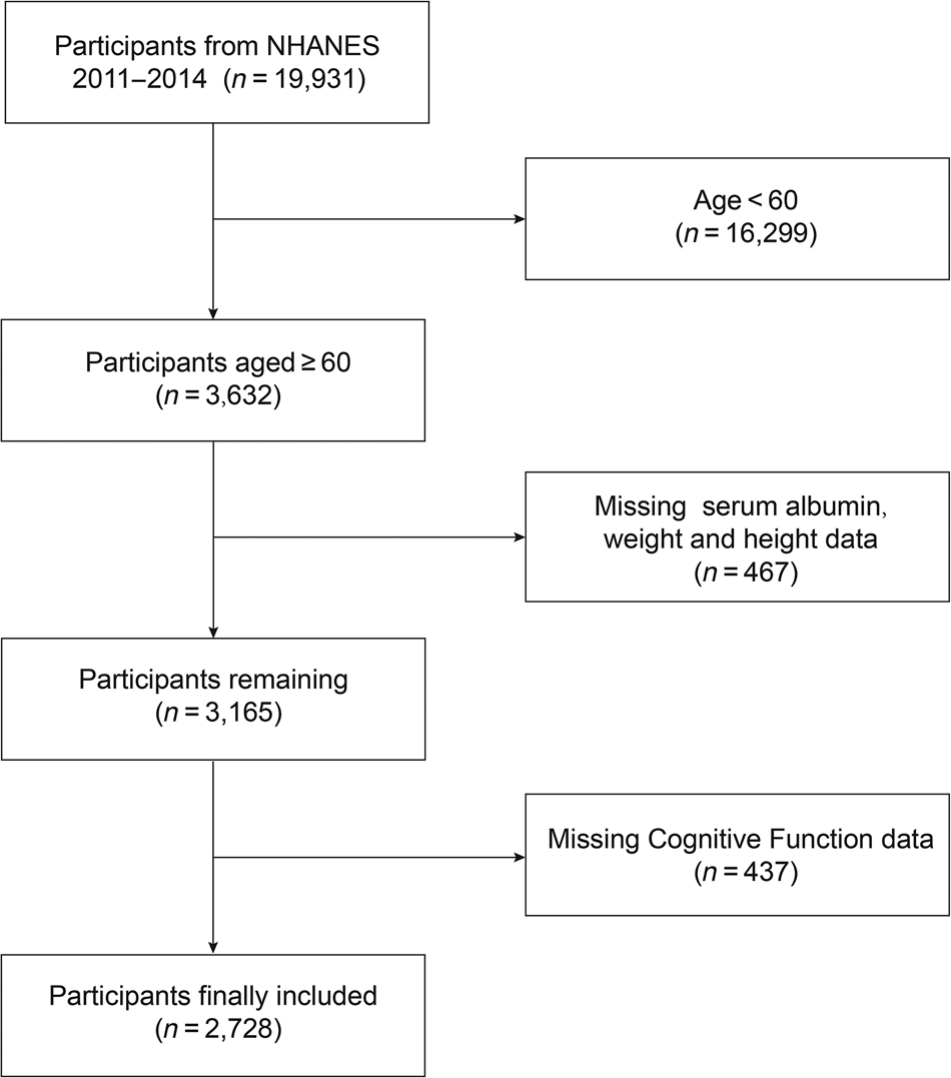
Flow chart of participants selection. NHANES, National Health and Nutrition Examination Survey.

This study utilized data from the National Health and Nutrition Examination Survey (NHANES), which gathers extensive demographic, dietary, physical, laboratory, and imaging information to assess the health and nutritional status of the US populace. NHANES employs a sophisticated, stratified, multistage probability sampling methodology, yielding a nationally representative sample of approximately 10,000 noninstitutionalized civilians annually (H. Tang et al., 2024; Wang et al., 2022). Participants from 2011 to 2012 and 2013 to 2014 were included in this study due to the availability of comprehensive cognitive function data. The research protocol secured approval from the National Center for Health Statistics (NCHS) Research Ethics Review Board. Furthermore, every participant engaged in the study provided informed written consent, ensuring adherence to ethical research standards. Initially, 19,931 participants from the specified NHANES cycles were identified. Following the exclusion of individuals under 60 years of age (16,299) and those lacking GNRI data (826) and cognitive function data (283), the final cohort comprised 2728 older adults (Figure 1).

### 2.2 Geriatric Nutritional Risk Index

The geriatric nutritional risk index was adapted from the Nutritional Risk Index (NRI) designed by Buzby et al. (1988). This index represents whole‐body nutritional status and is used to assess nutrition‐related complications. The GNRI is calculated using the formula: GNRI = 1.489 × serum albumin (g/L) + 41.7 × present weight/ideal weight (kg). Ideal body weight is determined by Lorentz's equations (Bouillanne et al., 2005), which are: for men, ideal weight = 0.75 × height (cm) − 62.5; for women, ideal weight = 0.60 × height (cm) − 40.

### 2.3 Measurement of cognitive function

To evaluate cognitive function in participants aged 60 and above, three distinct assessments were employed: The Consortium to Establish a Registry for Alzheimer's Disease Word Learning subtest (CERAD W‐L), the animal fluency test (AFT), and the digit symbol substitution test (DSST).

– CERAD W‐L evaluates memory function through an immediate recall test (IRT) and a subsequent delayed recall test (DRT) (Fillenbaum & Mohs, 2023). Participants memorize and recall a list of 10 unrelated words over three trials. The delayed word recall, assessing long‐term memory retention, occurs after the completion of the AFT and DSST, with each trial and the delayed recall total scored out of 10.

– AFT measures verbal skills, executive function, semantic memory, and the speed of thought processing (Canning et al., 2004). Participants are instructed to enumerate as many animal names as they can within a 1‐min timeframe, with each correctly named animal contributing one point to their overall score.

– DSST assesses mental agility, attention, visual scanning skills, and hand‐eye coordination (Campitelli et al., 2023). In this paper‐based test, participants match symbols to numbers based on a coding rule, filling 133 boxes within 2 min. The final score is determined by the total number of correct matches.

All of the above cognitive test scores have been standardized to facilitate better assessment of cognitive function. The global cognitive *z*‐scores are derived by averaging the standardized scores of the test scores of the CERAD–IRT, CERAD–DRT, AFT, and DSST.

### 2.4 Covariates

Covariate selection was informed by clinical insights and prior research findings (X.‐T. Huang et al., 2023; Li et al., 2024). The study included covariates such as gender (male or female), age (continuous, in years), race (categorized as Mexican American, other Hispanic, non‐Hispanic white, non‐Hispanic black, or other races), education level (less than high school, high school/GED (General Educational Development), or above high school), family poverty income ratio (PIR, low < 1.3, moderate 1.3–3.49, or high ≥ 3.5), Body Mass Index (BMI, kg/m^2^), drinking status (categorized as yes or no by more or less than 12 drinks per year), smoking status (categorized as yes or no by more or less than 100 cigarettes in a lifetime), and health conditions including diabetes, hypertension, dyslipidemia, stroke, cardiovascular disease and chronic kidney disease (each indicated as yes or no). The urinary albumin‐to‐creatinine ratio (ACR) was determined, utilizing an ACR > 30 mg/g as the criterion to identify renal disease presence. In addition, we included laboratory variables such as fasting blood glucose (mg/dL), glycated hemoglobin (%), total cholesterol (mg/dL), triglycerides (mg/dL), high‐density lipoprotein cholesterol (mg/dL), low‐density lipoprotein cholesterol (mg/dL), urine albumin (µg/mL), and urine creatinine (mg/dL). Comprehensive data on these variables are accessible through the public database of the NHANES (https://www.cdc.gov/nchs/nhanes/).

### 2.5 Statistical analysis

The statistical examination of the data was carried out using R software (version 4.3.2) and EmpowerStats (version 4.2). Adhering to NHANES protocols, the data were weighed and scrutinized accordingly. Continuous variables were articulated as mean ± standard deviation, and categorical variables were depicted in terms of frequencies or percentages. Descriptive analyses pivoted around GNRI quartiles, employing *t*‐tests and chi‐square tests to discern intergroup disparities. The relationship between cognitive function and GNRI was probed through multiple linear regression analyses. To isolate the GNRI's independent impact on cognitive function from other influencing factors, regression analyses were structured into three models. Model 1 was unadjusted; Model 2 was adjusted for gender, age, and race; and Model 3 made comprehensive adjustments for all covariates (gender, age, race, education, family PIR, BMI, drinking, smoking, health conditions, and laboratory variables). Moreover, generalized additive models and smooth curve fitting techniques were applied to explore the potential relationships between GNRI and cognitive function. Subgroup analyses and interaction tests were conducted based on gender, age, race, lifestyle, and health conditions to assess the GNRI‐cognitive function correlation across different population segments. In this analysis, a two‐sided *p* value of less than.05 was considered indicative of statistical significance.

## 3 RESULTS

### 3.1 Baseline characteristics

Our analysis encompassed 2728 participants, characterized by an average age of 69.42 ± 6.77 years, with a gender distribution of 48.97% males and 51.03% females. Table 1 delineates the baseline characteristics, categorized by GNRI quartiles. Notable disparities were observed across various demographic and health‐related variables, including age, gender, race, BMI, the status of diabetes, hypertension, and dyslipidemia (*p* < .05). The highest GNRI quartile predominantly comprised females, non‐Hispanic whites, and individuals with advanced education levels and medium income, alongside a trend of higher BMI and a lower prevalence of alcohol consumption. In addition, there was a positive association between elevated GNRI levels and an increased prevalence of diabetes and hypertension, as well as elevated urinary creatinine levels. Cognitive assessment scores—IRT, DRT, AFT, and DSST—displayed an upward trajectory in parallel with increasing GNRI levels, and global *z*‐scores similarly rose.

**TABLE 1:** Basic characteristics of participants by Geriatric Nutritional Risk Index (GNRI) quartiles among US older adults.

Characteristics	Quartile 1 (77.35–109.29)	Quartile 2 (109.30–115.87)	Quartile 3 (115.90–123.23)	Quartile 4 (123.24–212.39)	*p*‐value
**Number of participants**	682	682	682	682	
**Age (years)**	70.86 ± 7.19	69.68 ± 6.87	69.30 ± 6.65	67.82 ± 5.99	<.001
**Gender, *n* (%)**					<.001
Male	348 (51.03)	364 (53.37)	353 (51.76)	271 (39.74)	
Female	334 (48.97)	318 (46.63)	329 (48.24)	411 (60.26)	
**Race, *n* (%)**					<.001
Mexican American	34 (4.99)	63 (9.24)	72 (10.56)	75 (11.00)	
Other Hispanic	52 (7.62)	82 (12.02)	81 (11.88)	62 (9.09)	
Non‐Hispanic white	343 (50.29)	323 (47.36)	340 (49.85)	322 (47.21)	
Non‐Hispanic black	142 (20.82)	127 (18.62)	145 (21.26)	205 (30.06)	
Other races	111 (16.28)	87 (12.76)	44 (6.45)	18 (2.64)	
**Education level, *n* (%)**					.639
Less than high school	166 (24.34)	172 (25.22)	169 (24.78)	173 (25.37)	
High school or GED	152 (22.29)	159 (23.31)	157 (23.02)	178 (26.10)	
Above high school	364 (53.37)	351 (51.47)	356 (52.20)	331 (48.53)	
**Family PIR, *n* (%)**					.05
<1.31	191 (28.01)	163 (23.90)	177 (25.95)	211 (30.94)	
1.31−3.49	305 (44.72)	308 (45.16)	286 (41.94)	284 (41.64)	
≥3.50	186 (27.27)	211 (30.94)	219 (32.11)	187 (27.42)	
**BMI (kg/m^2^)**	22.93 ± 2.77	26.48 ± 2.27	29.78 ± 2.49	37.03 ± 5.71	<.001
**Drinking, *n* (%)**					.28
No	209 (30.65)	200 (29.33)	209 (30.65)	232 (34.02)	
Yes	473 (69.35)	482 (70.67)	473 (69.35)	450 (65.98)	
**Smoking, *n* (%)**					.454
No	323 (47.36)	330 (48.39)	348 (51.03)	346 (50.73)	
Yes	359 (52.64)	352 (51.61)	334 (48.97)	336 (49.27)	
**Diabetes, *n* (%)**					<.001
No	534 (78.30)	498 (73.02)	449 (65.84)	378 (55.43)	
Yes	148 (21.70)	184 (26.98)	233 (34.16)	304 (44.57)	
**Hypertension, *n* (%)**					<.001
No	256 (37.54)	217 (31.82)	193 (28.30)	143 (20.97)	
Yes	426 (62.46)	465 (68.18)	489 (71.70)	539 (79.03)	
**Dyslipidemia, *n* (%)**					<.001
No	347 (50.88)	300 (43.99)	265 (38.86)	287 (42.08)	
Yes	335 (49.12)	382 (56.01)	417 (61.14)	395 (57.92)	
**Stroke, *n* (%)**					.447
No	628 (92.08)	641 (93.99)	636 (93.26)	641 (93.99)	
Yes	54 (7.92)	41 (6.01)	46 (6.74)	41 (6.01)	
**CVD, *n* (%)**					.331
No	567 (83.14)	565 (82.84)	574 (84.16)	549 (80.50)	
Yes	115 (16.86)	117 (17.16)	108 (15.84)	133 (19.50)	
**CKD, *n* (%)**					.054
No	544 (79.77)	580 (85.04)	549 (80.50)	552 (80.94)	
Yes	138 (20.23)	102 (14.96)	133 (19.50)	130 (19.06)	
Fast glucose (mg/dL)	108.01 ± 27.01	108.85 ± 23.84	110.40 ± 23.79	113.93 ± 27.95	<.001
Glycohemoglobin (%)	5.91 ± 1.09	6.00 ± 1.11	6.12+ 1.05	6.25 ± 1.07	<.001
Total cholesterol (mg/dL)	190.20 ± 43.78	194.14 ± 42.75	192.73 ± 44.45	188.89 ± 41.78	.086
Triglyceride (mg/dL)	102.31 ± 43.46	112.36 ± 49.10	116.01 ± 49.88	124.76 ± 59.95	<.001
High‐density lipoprotein cholesterol (mg/dL)	59.68 ± 18.06	55.25 ± 16.21	51.85 ± 16.01	50.76 ± 13.03	<.001
Low‐density lipoprotein cholesterol (mg/dL)	110.04 ± 25.85	109.87 ± 25.53	109.08 ± 25.22	107.71 ± 25.76	.623
Urine albumin (µg/mL)	80.65 ± 388.75	39.91 ± 189.91	66.28 ± 290.72	68.77 ± 379.72	.023
Urine creatinine (mg/dL)	101.12 ± 64.69	107.86 ± 69.36	110.54 ± 69.42	119.41 ± 71.88	<.001
**Cognitive score**					
CERAD–IRT	18.43 ± 4.97	18.86 ± 4.59	19.11 ± 4.43	19.50 ± 4.39	.004
CERAD–DRT	5.74 ± 2.45	5.81 ± 2.32	5.98 ± 2.26	6.29 ± 2.19	<.001
AFT	15.98 ± 5.46	16.64 ± 5.69	16.96 ± 5.22	17.17 ± 5.41	<.001
DSST	44.39 ± 17.69	46.18 ± 17.38	47.28 ± 16.83	46.77 ± 16.83	.007
Global *z*‐score	−0.11 ± 0.83	−0.02 ± 0.79	0.04 ± 0.75	0.10 ± 0.75	<.001

*Note*: Mean  ±  SD for continuous variables: the *p* value was calculated by the weighted linear regression model; (%) for categorical variables: the *p* value was calculated by the weighted chi‐square test.

Abbreviations: GED, General Educational Development; AFT, animal fluency test; BMI, body mass index; CERAD, Consortium to Establish a Registry for Alzheimer's Disease; CKD, chronic kidney disease; CVD, cardiovascular disease; DRT, delayed recall test; DSST, digit symbol substitution test; IRT, immediate recall test; PIR, poverty income ratio.

### 3.2 Association between GNRI and cognitive function

Upon dividing participants into quartiles based on their GNRI levels, we observed notable enhancements in IRT scores, DRT scores, AFT scores, DSST scores, and global z‐scores, corresponding to higher GNRI quartiles (Table 2). This trend persisted across all analytical models. Specifically, Model 1 demonstrated a significant link between higher GNRI levels and augmented cognitive function global *z*‐scores, a pattern that held steady in the more rigorously adjusted Model 3 (*p* for trend < .01).

**TABLE 2:** Associations between Geriatric Nutritional Risk Index (GNRI) and cognitive function.

Cognition score	Model 1 (*β* [95% CI])	Model 2 (*β* [95% CI])	Model 3 (*β* [95% CI])
**CERAD–IRT**	0.04 (0.02, 0.05)	0.01 (−0.00, 0.03)	0.04 (0.01, 0.08)
Q1	Reference	Reference	Reference
Q2	0.43 (−0.06, 0.92)	0.36 (−0.10, 0.82)	0.35 (−0.13, 0.82)
Q3	0.68 (0.19, 1.17)	0.50 (0.04, 0.97)	0.55 (0.00, 1.09)
Q4	1.07 (0.58, 1.55)	0.38 (−0.09, 0.85)	0.59 (−0.19, 1.36)
*p* for trend	<.0001	.1236	.0255
**CERAD–DRT**	0.02 (0.01, 0.03)	0.01 (0.00, 0.02)	0.02 (−0.00, 0.04)
Q1	Reference	Reference	Reference
Q2	0.07 (−0.18, 0.31)	0.03 (−0.19, 0.26)	0.02 (−0.22, 0.27)
Q3	0.23 (−0.01, 0.48)	0.19 (−0.04, 0.42)	0.19 (−0.08, 0.47)
Q4	0.55 (0.30, 0.79)	0.30 (0.07, 0.54)	0.34 (−0.05, 0.73)
*p* for trend	<.0001	.0073	.0765
**AFT**	0.04 (0.02, 0.05)	0.02 (−0.00, 0.03)	0.06 (0.02, 0.11)
Q1	Reference	Reference	Reference
Q2	0.66 (0.08, 1.24)	0.42 (−0.12, 0.97)	0.47 (−0.08, 1.03)
Q3	0.98 (0.40, 1.55)	0.56 (0.02, 1.11)	0.72 (0.09, 1.35)
Q4	1.18 (0.61, 1.76)	0.58 (0.02, 1.13)	1.10 (0.20, 1.99)
*p* for trend	<.0001	.068	.0043
**DSST**	0.09 (0.03, 0.14)	0.03 (−0.02, 0.08)	0.17 (0.06, 0.28)
Q1	Reference	Reference	Reference
Q2	1.79 (−0.04, 3.61)	1.75 (0.20, 3.30)	1.34 (−0.06, 2.75)
Q3	2.89 (1.07, 4.71)	2.72 (1.16, 4.28)	2.55 (0.94, 4.16)
Q4	2.38 (0.55, 4.20)	0.81 (−0.79, 2.40)	1.57 (−0.71, 3.86)
*p* for trend	.0013	.272	.0019
**Global *z*‐score**	0.01 (0.00, 0.01)	0.00 (0.00, 0.01)	0.01 (0.00, 0.01)
Q1	Reference	Reference	Reference
Q2	0.09 (0.00, 0.17)	0.07 (−0.00, 0.14)	0.06 (−0.01, 0.13)
Q3	0.15 (0.07, 0.23)	0.11 (0.04, 0.19)	0.12 (0.04, 0.20)
Q4	0.21 (0.12, 0.29)	0.09 (0.02, 0.17)	0.14 (0.03, 0.26)
*p* for trend	<.0001	.0167	.0007

*Note*: Model 1: no covariates were adjusted. Model 2: adjusted for gender, age, and race. Model 3: adjusted for gender, age, race, education level, family PIR, BMI, drinking, smoking, diabetes, hypertension, dyslipidemia, CVD, CKD, fast glucose, glycated hemoglobin, total cholesterol, triglycerides, high‐density lipoprotein cholestero, low‐density lipoprotein cholestero, urine albumin, and urine creatinine.

**FIGURE 2:**
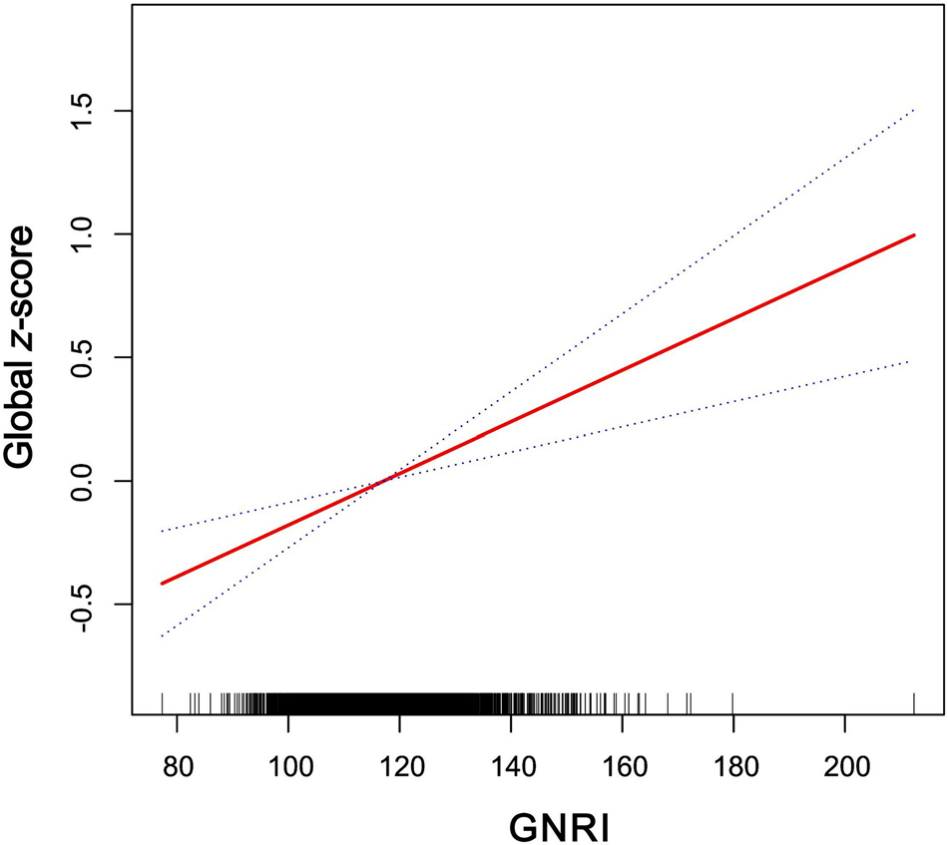
Association between Geriatric Nutritional Risk Index (GNRI) and cognitive function. The solid red line represents the smooth curve fit between variables. Blue bands represent the 95% of confidence interval from the fit. Adjusted for gender, age, race, education level, family poverty income ratio (PIR), body mass index (BMI), drinking, smoking, diabetes, hypertension, dyslipidemia, cardiovascular disease (CVD), chronic kidney disease (CKD), fast glucose, glycated hemoglobin, total cholesterol, triglycerides, high‐density lipoprotein cholesterol, low‐density lipoprotein cholestero, urine albumin, and urine creatinine.

**FIGURE 3:**
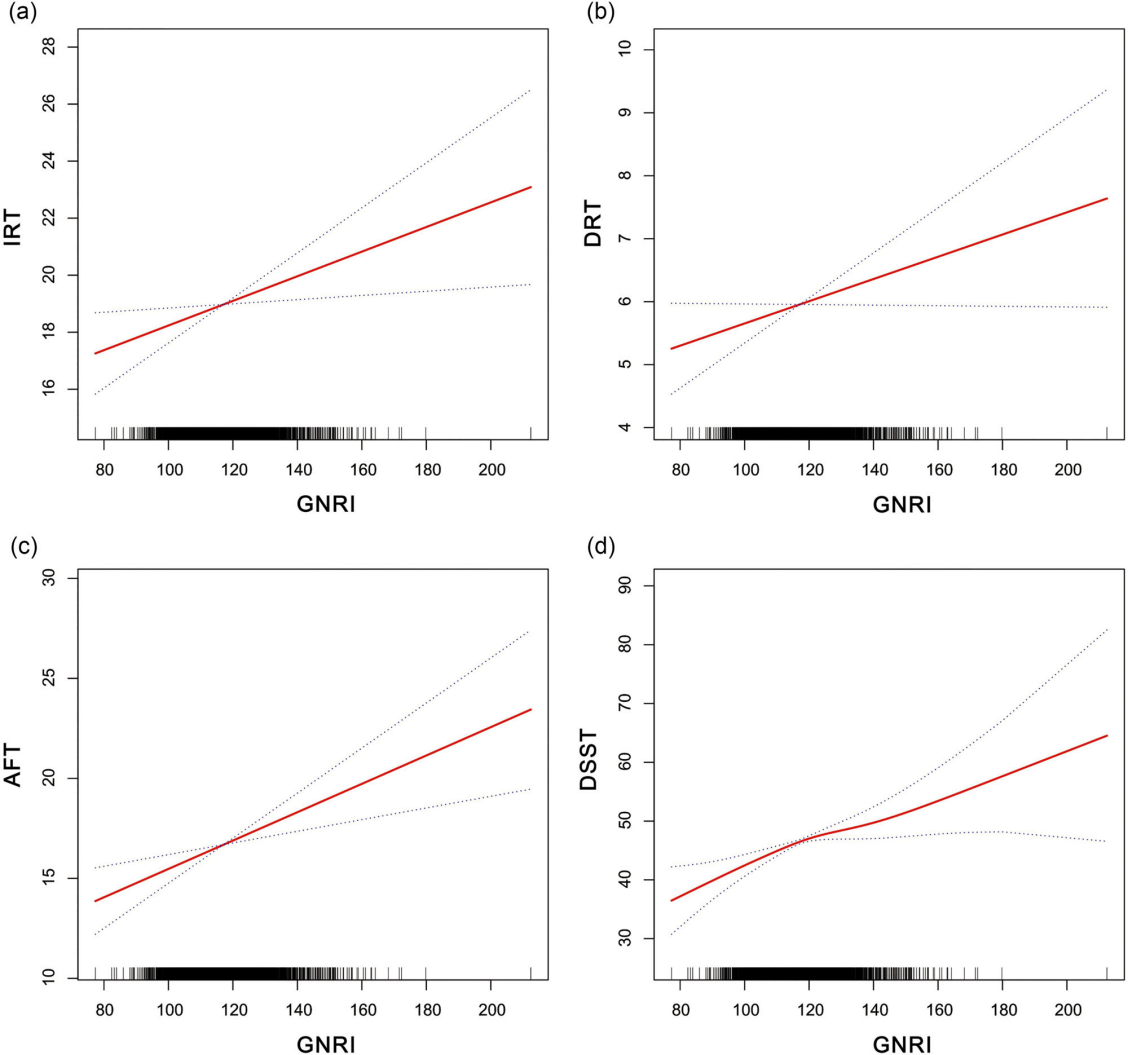
Association between Geriatric Nutritional Risk Index (GNRI) and cognitive function and components. (a) GNRI and Consortium to Establish a Registry for Alzheimer's Disease (CERAD) immediate recall test; (b) GNRI and CERAD delayed recall test; (c) GNRI and animal fluent test; (d) GNRI and digit symbol substitution test. AFT, animal fluency test; DRT, delayed recall test; DSST, digit symbol substitution test; IRT, immediate recall test.

Moreover, after adjusting for all confounders, the smoothed curve fitting results showed a linear relationship between GNRI and cognitive function in older adults, further reinforcing the positive correlation (Figure 2). Meanwhile, Figure 3 shows that scores on all four cognitive tests increased significantly with increasing levels of GNRI. The linear relationship between GNRI and IRT, DRT, and AFT remained.

To examine the consistency of the relationship between GNRI and cognitive function in the overall population and to identify potential differences between specific populations, we performed subgroup analyses and interaction tests by gender, age, race, and status of drinking, smoking, diabetes, hypertension, dyslipidemia, stroke, CVD, and CKD. Table 3 shows that the positive association between GNRI and cognitive function remained significant across subgroups, including gender, age, smoking, diabetes, and hypertension. Subsequently, we found a significant positive correlation between GNRI and cognitive functioning in individuals with a BMI of <25, with a 0.09 increase in global *z*‐scores (*β* = 0.09; 95% confidence interval [CI] = 0.01, 0.16) for each unit increase in GNRI. In addition, no significant interactions were found between any of the stratification parameters, suggesting that the association between GNRI and cognitive function is independent of gender, age, race, BMI, lifestyle, and health conditions (*p* for interaction > .05).

**TABLE 3:** Subgroup analysis of the association between Geriatric Nutritional Risk Index (GNRI) and cognitive function.

Subgroup	Total	*β* (95% CI)	*p* for interaction
Gender			0.8792
Male	1336	0.09 (0.03, 0.15)	
Female	1392	0.10 (0.04, 0.15)	
Age (years)			0.7804
<70	1488	0.11 (0.03, 0.18)	
≥70	1240	0.12 (0.04, 0.20)	
Race/ethnicity			0.50404
Mexican American	244	0.15 (−0.03, 0.33)	
Other Hispanic	277	0.15 (−0.02, 0.31)	
Non‐Hispanic white	1328	0.11 (0.04, 0.19)	
Non‐Hispanic black	619	0.04 (−0.06, 0.15)	
Other races	260	−0.02 (−0.20, 0.17)	
BMI (kg/m^2^)			0.2857
<25	735	0.09 (0.01, 0.16)	
25–29.9	966	0.07 (−0.01, 0.15)	
≥30	1027	0.03 (−0.01, 0.06)	
Drinking			0.5535
No	850	0.07 (−0.03, 0.17)	
Yes	1878	0.10 (0.04, 0.17)	
Smoking			0.649
No	1347	0.08 (0.00, 0.16)	
Yes	1381	0.11 (0.03, 0.18)	
Diabetes			0.2571
No	1859	0.07 (0.00, 0.14)	
Yes	869	0.14 (0.04, 0.23)	
Hypertension			0.7764
No	809	0.11 (0.00, 0.21)	
Yes	1919	0.09 (0.03, 0.15)	
Dyslipidemia			0.0691
No	1199	0.15 (0.07, 0.22)	
Yes	1529	0.05 (−0.02, 0.12)	
Stroke			0.5861
No	2546	0.09 (0.03, 0.15)	
Yes	182	0.14 (−0.04, 0.33)	
CVD			0.3977
No	2255	0.10 (0.05, 0.16)	
Yes	473	0.04 (−0.09, 0.17)	
CKD			0.7885
No	2225	0.10 (0.04, 0.16)	
Yes	503	0.08 (−0.04, 0.20)	

*Note*: Adjusted for all covariates except effect modifier.

## 4 DISCUSSION

In this cross‐sectional study exploring the association between GNRI and cognitive functioning in older adults, our study encompassed 2728 older Americans. We found a significant positive association between increased GNRI levels and cognitive health, with and without adjustment for covariates. These results align with our initial hypothesis. Further subgroup analyses, which considered renal function status alongside various demographic and lifestyle factors, confirmed that the positive correlation remained robust across different groups. This consistency suggests that the relationship between higher GNRI and enhanced cognitive function is likely applicable to a diverse range of populations, regardless of variations in renal function, gender, age, race, BMI, and lifestyle habits. Based on these findings, we advocate for the improvement of GNRI as a complementary approach within the multifactorial management of cognitive health in older adults. Enhancing nutritional status through strategies that elevate GNRI could serve as an effective measure to boost cognitive function and overall well‐being in this demographic.

Previous findings on the correlation between poorer cognitive functioning in older adults and serum albumin levels and obesity‐related parameters are controversial. A longitudinal study by Dik et al. (2005) found that after administering the Mini‐Mental State Examination (MMSE), Auditory Verbal Learning Test, Raven's Colored Progressive Matrices, and coding task tests to 1284 older participants, the results showed that serum albumin was not associated with cognitive decline on any of the cognitive tests. However, a recent population‐based Chinese longitudinal health longevity survey (CLHLS) study, which included 2017 older adults aged 65 years and older and measured cognitive function using the Mini‐Mental State Examination (MMSE), provided evidence that serum albumin levels were inversely associated with the risk of cognitive impairment in older adults (Yin et al., 2016). This observation was supported by Santulli et al. (2024), who observed a significant negative correlation between albuminuria and cognitive decline in a frail elderly population. In addition, a cohort study in the United States also noted that serum albumin identifies individuals with Parkinson's disease who are at risk for cognitive decline (Shen et al., 2022). Previous studies have shown that lower BMI is associated with better cognitive function (Liu et al., 2019), while obesity is associated with poorer cognitive function in older adults (X. Tang et al., 2021). However, the study by Luchsinger et al. (2013) showed contradictory results, with higher rates of obesity and higher fat‐free mass in older adults being associated with better cognitive function. This was confirmed by a cross‐sectional study in Singapore, where low BMI combined with chronic disease was independently associated with poor cognitive performance in community‐dwelling older adults (Ng et al., 2008). Therefore, GNRI may be a more reliable indicator for detecting cognitive function in older adults than serum albumin levels and obesity‐related parameters. This is consistent with the findings of the present study, which found that GNRI was positively associated with cognitive health and that this relationship was independent of factors such as age, gender, race, smoking, drinking, and health conditions.

The GNRI serves as an important assessment tool that combines three metrics: serum albumin, body weight, and ideal body weight, with the role of serum albumin being particularly critical. Low serum albumin is often seen in nephrotic syndrome and malnourished chronic kidney disease. Serum albumin is not only an indicator of nutritional status; it also serves a variety of physiological functions, including maintaining plasma colloid osmolality, transporting drugs and hormones (Rimac et al., 2017), and acting as an antioxidant. Low levels of serum albumin have been associated with brain atrophy, neuronal damage, and neuroinflammation (Deng et al., 2021), all of which are important biological mechanisms for cognitive decline. Serum albumin acts as an antioxidant, neutralizing free radicals and reducing damage to nerve cells from oxidative stress (Belinskaia et al., 2021). Oxidative stress is considered a key contributor to neurodegenerative diseases such as Alzheimer's disease (Bai et al., 2022) and Parkinson's disease (Raza et al., 2019). Notably, studies have shown that SGLT2‐inhibitors can enhance cognitive function in elderly diabetic patients with chronic kidney disease by improving metabolism and reducing oxidative stress (Mone et al., 2024). Oxidative stress also significantly contributes to both cognitive and physical decline in frail older adults, and nutritional interventions can help mitigate these effects (Mone et al., 2023). Moreover, low serum albumin levels often reflect inadequate protein intake or nutrient absorption issues, leading to insufficient nutritional support for the brain and thus impairing cognitive function (Hyman, 2005). It is worth noting that low levels of serum albumin are associated with a state of chronic inflammation (Sheinenzon et al., 2021), which is an important factor in cognitive decline. The inflammatory response can directly damage nerve cells by promoting abnormal deposition of amyloid and tau proteins, affecting the function and structure of the brain (Tzioras et al., 2023). It has been shown that β‐amyloid deposition is an important factor in the development of Alzheimer's disease and cognitive impairment (Jagust et al., 2023), and serum albumin can bind to β‐amyloid and is an important regulator of β‐amyloid clearance (Kim et al., 2020). Weight and ideal body weight are additional important indicators within GNRI, helping to reflect an individual's nutritional status more accurately. Weight loss is often seen as a sign of malnutrition, especially if weight loss is not intentional (Zonneveld et al., 2023). Although height in older adults may be reduced due to decreased bone density and spinal curvature associated with aging, incorporating ideal body weight when assessing the GNRI can help to more accurately reflect an individual's nutritional status and physical health (Bouillanne et al., 2005). The GNRI provides a comprehensive tool for assessing the nutritional status and cognitive health of older adults by combining serum albumin, body weight, and ideal body weight. Good nutritional status is an important factor in maintaining cognitive function and preventing neurodegenerative diseases. By monitoring GNRI, physicians can identify malnourished older adults, especially those with chronic kidney disease, and take appropriate interventions, such as supplementation and dietary modifications, to improve their nutritional status and cognitive health.

This study's strength emanates from employing NHANES data, ensuring a large, nationally representative sample through stratified multistage probability sampling. We meticulously adjusted for potential confounders, including lifestyle and health conditions, thereby bolstering the credibility of our results. Subgroup analyses further scrutinized the robustness of the GNRI‐cognitive function correlation across diverse populations. It is noteworthy that this study is the first to explore the correlation between GNRI and cognitive function in older adults. However, the study is not devoid of limitations. Given the cross‐sectional design of this investigation, establishing causality between GNRI and cognitive performance in the elderly remains elusive. It remains ambiguous whether an elevated GNRI actively contributes to cognitive well‐being or if cognitive decline precipitates nutritional deficiencies. Furthermore, this research relied exclusively on data from the NHANES, incorporating nutritional assessments and cognitive evaluations captured at a singular moment, potentially not capturing the longitudinal nutritional and cognitive trajectory of individuals. Therefore, large‐scale prospective studies are necessary to fully understand and apply the results of this study.

## 5 CONCLUSION

Our study concluded that there is a positive association between GNRI levels and cognitive health in older adults and that this association is consistent across CKD and non‐CKD populations. These findings emphasize that nutritional status may serve as an important component in identifying and treating cognitive impairment in older adults.

## AUTHOR CONTRIBUTIONS

**Shan Wang**: Conceptualization; data curation; methodology; software; writing—original draft. **Jiajia Zhang**: Data curation; validation; writing—original draft. **Jiaru Zhuang**: Data curation. **Yuan Wang**: Investigation. **Dewu Xu**: Supervision; writing—review and editing. **Yibo Wu**: Conceptualization; writing—review and editing.

## CONFLICT OF INTEREST STATEMENT

The authors declare no conflicts of interest.

### PEER REVIEW

The peer review history for this article is available at https://publons.com/publon/10.1002/brb3.70015

## Data Availability

This study made use of publicly available datasets, with all the data pertinent to our research accessible via the official National Health and Nutrition Examination Survey website: https://www.cdc.gov/nchs/nhanes/.
